# Mycophenolate Interruption Restores Anti-SARS-CoV-2 Vaccine Immunogenicity in Unresponsive Liver Transplant Recipients

**DOI:** 10.3390/vaccines11071165

**Published:** 2023-06-27

**Authors:** Pierluigi Toniutto, Annarosa Cussigh, Sara Cmet, Martina Fabris, Francesco Curcio, Davide Bitetto, Ezio Fornasiere, Elisa Fumolo, Edmondo Falleti

**Affiliations:** 1 Hepatology and Liver Transplantation Unit, Department of Specialized Medicine, Udine University Hospital, 33100 Udine, Italy; davide_bitetto@yahoo.it (D.B.); eziofornasiere@hotmail.com (E.F.); e.fumolo@hotmail.it (E.F.); edmondofalleti@libero.it (E.F.); 2 Clinical Pathology, Udine University Hospital, 33100 Udine, Italy; annarosa.cussigh@asufc.sanita.fvg.it (A.C.); sara.cmet@asufc.sanita.fvg.it (S.C.); martina.fabris@asufc.sanita.fvg.it (M.F.); francesco.curcio@uniud.it (F.C.)

**Keywords:** SARS-CoV-2 infection, mycophenolate mofetil, liver transplantation, anti-SARS-CoV-2 vaccine

## Abstract

Background & aims: The fourth dose of anti-SARS-CoV-2 vaccine slightly improved the humoral response among previously seronegative liver transplant (LT) recipients. Mycophenolate (MMF) treatment worsens the vaccination response. This study aimed to evaluate whether temporary MMF interruption might improve the immunogenicity of the fourth anti-SARS-CoV-2 BNT16b2 vaccine dose in nonresponsive LT recipients. Methods: LT recipients negative for anti-spike glycoprotein-specific immunoglobulin G receptor-binding domain (s-RBD) antibodies after the third vaccine dose were enrolled. Anti-SARS-CoV-2 spike-specific T-cell responses were measured before and 2 months following the fourth vaccine dose, and anti-SARS-CoV-2 s-RBD antibodies also 6 months thereafter. MMF was suspended two weeks before and after vaccination. Results: Five LT recipients were enrolled. After a mean of 78 days after vaccination, all patients tested positive for anti-SARS-CoV-2 s-RBD antibodies. The mean antibody titer was 8944 UI/mL. The positive antibody response was maintained during a mean of 193 days of follow-up. Three patients developed a positive T-cell response. Two patients (one positive for T-cell response) developed a self-limited SARS-CoV-2 infection. Conclusions: Suspending MMF prior to the fourth dose of the anti-SARS-CoV-2 mRNA vaccine seems feasible and safe. This procedure could restore vaccine-induced immunogenicity in a large portion of previously nonresponsive LT recipients.

## 1. Introduction

Vaccinations play a crucial role in protecting individuals from infections, especially those who are immunocompromised. These patients have weakened immune systems, which can make them more susceptible to several kinds of infections [1]. Thus, in immunocompromised patients, including those with advanced liver disease and those who underwent a liver transplant (LT), vaccinations are encouraged by several scientific societies [2,3]. Although in patients with advanced liver diseases, all vaccinations are recommended, in LT recipients, live-attenuated vaccines, such as measles, mumps, rubella, varicella-zoster, herpes-zoster, yellow fever, and tuberculosis, are contraindicated, given the risk of an active vaccine-induced infection [2,3]. Despite the widespread use of vaccinations in patients with advanced liver disease and in LT recipients, it has become evident that the immunogenicity of many vaccines is significantly lower than that observed in immunocompetent patients [4]. This is true, for example, for vaccinations against hepatitis B (HBV), hepatitis A (HAV), and flu viruses [5,6]. However, it still remains unclear if a specific type of immunosuppression schedule or a single immunosuppressive agent, such as mycophenolate (MMF), could be responsible for a decreased immunogenicity of many kinds of vaccination in these patients. Until now, no studies have been performed to evaluate the potential benefit of reducing immunosuppressive treatments in increasing the immunogenicity of several kinds of vaccines in solid-organ-transplanted (SOT) recipients.

The recent acute respiratory syndrome coronavirus-2 (SARS-CoV-2) epidemic highlighted this challenging issue with respect to vaccination for this disease.

Vaccines against severe SARS-CoV-2 in LT recipients induced decreased immunogenicity compared to the general population [7,8]. Although the administration of a booster (third) dose of mRNA-based vaccines increased the immune response rates produced by 2 vaccine doses by 20% [9], up to 30% of LT patients remained unresponsive to the vaccination [7,8]. Thus, the administration of a fourth dose of anti-SARS-CoV-2 vaccines seems to be a useful way to increase immunogenicity in LT and in SOT recipients [10]. It has been demonstrated that MMF plays a major role in reducing the probability of achieving positive antibody [9,11] and T-cell [12] immune responses following a booster dose of mRNA-based anti-SARS-CoV-2 vaccines in LT recipients and in patients with autoimmune diseases [13]. This could be explained by decreases in lymphocyte activation, the interaction of T cells with antigen-presenting cells, and B-cell memory responses caused by prolonged administration of MMF [14]. Despite this evidence, still no data are available regarding the benefit of MMF interruption in increasing humoral and T-cell-mediated immune responses after the fourth dose of anti-SARS-CoV-2 vaccine in nonresponsive LT recipients.

The current study, involving five patients, is a pilot study that explored if temporary interruption of MMF might improve humoral and T-cell-mediated immune responses after the fourth dose of anti-SARS-CoV-2 BNT16b2 vaccine in previously nonresponsive LT recipients. Furthermore, the incidence of symptomatic coronavirus-19 disease (COVID-19) was evaluated up to 6 months after the fourth vaccination dose.

## 2. Materials and Methods

### 2.1. Ethics Statement

All patients provided written informed consent to receive the fourth vaccine dose and to participate in this study, which conformed to the ethical guidelines of the Declaration of Helsinki as revised in 2013. The evaluation of the anonymized patient data was approved by the Ethical Committee of the Academic Hospital of Udine, where the study was performed.

### 2.2. Study Design and Selection of Patients

Patients enrolled were selected from a recently reported cohort of nonresponsive LT recipients who completed three doses of the anti-SARS-CoV-2 BNT162b2 vaccine [9]. All patients were taking MMF, and the fourth SARS-CoV-2 BNT16b2 vaccine dose was offered at a mean (range) of 226 (174–273) days following the third dose. Anti-SARS-CoV-2-N protein IgM and IgG (iFlash^®^—Shenzhen Yhlo Biotech Co., Ltd., Shenzhen, China) and anti-spike glycoprotein-specific immunoglobulin G receptor-binding domain (s-RBD) antibodies (Roche Elecsys^®^, F. Hoffmann-La Roche Ltd., Basel, Switzerland) were measured before and twice between 2 and 6 months after the fourth vaccine dose. Following the manufacturer’s instructions and previously published reports [9], the cut-off values used to identify positive patients for the anti-SARS-CoV-2 N and s-RBD protein antibodies were >10.0 kAU/L and ≥0.8 U/mL, respectively. The anti-SARS-CoV-2 spike-specific T-cell response was assessed by the interferon-γ release assay (Euroimmun Diagnostica, Padova, Italy), both at baseline and two months after the fourth vaccine dose. The T-cell response was considered positive if interferon-γ was measured at ≥12 pg/mL above the negative control.

An expert team of transplant hepatologists selected patients in which to propose the MMF interruption two weeks before and after the fourth vaccine dose administration. Patients who experienced acute or chronic cellular rejection or poor adherence to immunosuppressive treatment were excluded. Furthermore, patients who could not carry out close and frequent clinical checks and the evaluation of laboratory parameters concerning the function of the liver graft and the measurement of plasma levels of immunosuppressive drugs were excluded. During the MMF interruption period, tacrolimus monotherapy was maintained with a mean (range) serum level of 4.82 (3.4–6.49) ng/mL, and liver function tests were evaluated twice a month, up to 3 months after the vaccination.

## 3. Results

Among the 107 LT recipients from the original cohort previously described [9], 9 (8.4%) were identified as vaccine nonresponsive. Two patients refused the fourth vaccine dose, and two tested positive for a past SARS-CoV-2 infection. Thus, five patients were finally included in the present study. The main clinical and demographic patient characteristics are reported in Table 1. The majority of patients were males, and the mean (range) time from LT to the fourth vaccine dose was 94 (19–314) months. All patients presented an optimal liver graft function, as demonstrated by the normality of transaminases and albumin serum levels. The etiology of liver disease was viral in two cases, autoimmune in two cases, and alcoholic in the remaining case.

**Figure 1:**
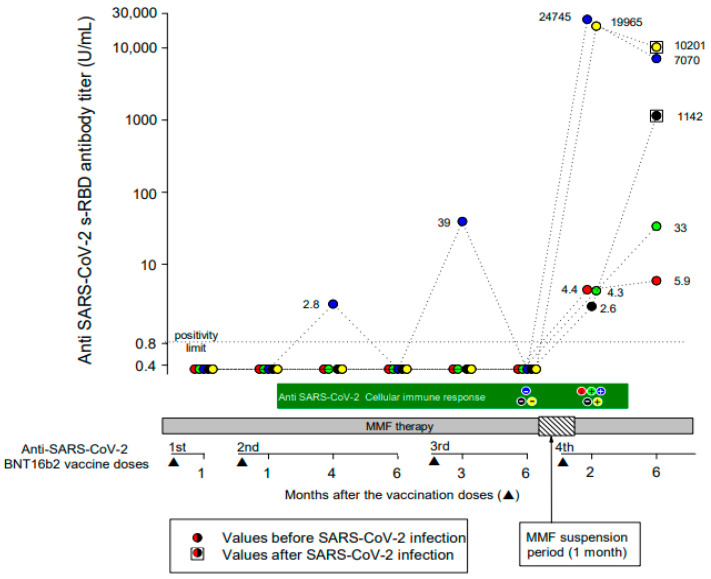
Anti-SARS-CoV-2 s-RBD IgG antibody and T-cell responses during the full course of four doses of the BNT162b2 vaccine. Each colored circle identifies a patient, as represented in Table 1. Circles within squares identify patients who developed symptomatic SARS-CoV-2 infection during the follow-up period.

After a mean (range) time of 78 (62–113) days after the fourth vaccine dose, all patients tested positive for IgG anti-SARS-CoV-2 s-RBD antibodies. The mean (range) anti-SARS-CoV-2 s-RBD antibody titer was 8944 (2.6–24,745) UI/mL. A total of 2 patients developed a strong and 3 a weak (mean antibody titer of 22,355 and 3.8 U/mL, respectively) antibody response. No demographic or clinical differences between strong and weak responders were evident. A further anti-SARS-CoV-2 s-RBD antibody titer was measured at a mean (range) time of 193 (140–245) days after the fourth vaccine dose. All patients maintained a positive antibody response to vaccination, but the mean (range) antibody titer decreased to 3690 (5.9–10,201) U/mL (Figure 1).

Measurements of the vaccine-induced SARS-CoV-2 spike-specific T-cell response were available at the time of the fourth vaccine dose in 3/5 patients and were negative in all patients. At 2 months after the fourth vaccine dose, 3/5 patients (1 negative before the fourth vaccine dose) developed a positive T-cell immune response, while 2 patients remained negative (Figure 1). Interestingly, 2/3 patients who developed a positive T-cell immune response presented the highest anti-SARS-CoV-2 s-RBD IgG antibody titers both at 2 months and up to 6 months after the fourth vaccine dose.

Two male patients developed symptomatic COVID-19 three and four months after the fourth vaccine dose, respectively. The anti-SARS-CoV-2 s-RBD antibody titer, measured at the time of COVID-19 resolution and compared to that recorded 2 months after the vaccine dose, increased from 2.6 U/mL to 1142 U/mL in 1 patient and decreased from 19,965 U/mL to 10,201 U/mL in the other (Figure 1). In 1 patient, the T-cell immune response was negative both before and after the fourth vaccine dose, and he presented a low (2.6 U/mL) anti-SARS-CoV-2 s-RBD antibody titer 2 months after the fourth vaccine dose. In the other patient, the T-cell immune response was negative only before the fourth vaccine dose, and he presented a very high (19,965 U/mL) antibody titer 2 months after the fourth vaccine dose.

The clinical course of COVID-19 in these two patients was mild, and neither of them required hospital admission. No severe side effects of vaccination were recorded, and liver function tests remained unchanged during the entire follow-up.

## 4. Discussion

To our knowledge, this is the first study that evaluated the impact of temporary MMF interruption on both humoral and T-cell immune responses following the fourth BNT162b2 vaccine dose in previously nonresponsive LT patients. The decision to temporarily suspend MMF could be controversial, as it could increase the risk of rejection. However, the duration of MMF suspension was very short and was adopted more than 1.5 years after LT. After this period, the clinical guidelines suggest that patients can be maintained on tacrolimus monotherapy with target serum levels near 5 mg/mL [15], as reported in our study.

All enrolled patients developed a positive antibody response, and 3/5 patients developed a positive T-cell immune response after the fourth vaccine dose. The positive impact of MMF interruption in increasing the vaccine-induced immunogenicity provided by our study was demonstrated in a case series of patients with rheumatic and musculoskeletal diseases [16], but not in solid organ transplant recipients, such as kidney-transplanted patients [17]. These different results could depend on the shorter MMF interruption period adopted in kidney transplant recipients (2 weeks) compared to our patients. Our data seem to be in agreement with those recently reported in a group of 10 LT patients nonresponsive to the third anti-SARS-CoV-2 vaccine dose, in whom MMF administration was interrupted before the fourth vaccine dose [18]. All patients presented positive anti-spike IgG antibodies following 2 to 4 weeks after the fourth vaccine dose. However, it is important to note that four patients were already responsive, albeit with a low antibody titer, to the third vaccination, thus the final percentage of vaccine-responsive patients could be overestimated. Moreover, the antibody titer was measured only 4 weeks after the fourth vaccine dose, and the T-cell response was not evaluated in this study.

Two patients in our study developed symptomatic COVID-19, despite a positive antibody response to the fourth vaccine dose, and one patient developed symptomatic COVID-19, despite a positive T-cell immune response. A recent report indicated that 10% of SOT recipients vaccinated with 4 doses developed COVID-19 during the Omicron wave [19,20]. Interestingly, it seems that the fourth vaccine dose failed to induce significant neutralization of the Omicron variant in SOT, indicating that 10- to 20-fold higher titers are required to neutralize this heavily mutated variant in vitro [20]. These findings suggest that additional dosing with the original vaccines in SOT recipients may not generate robust protection against infection in the form of neutralizing antibodies against the Omicron variant or future variants evolved from Omicron.

Our study has some limitations. The number of patients enrolled was small, but no other studies with the same study design have been reported so far. Furthermore, neutralizing antibody measurements and the characterization of SARS-CoV-2 variants in the two breakthrough infections were not evaluated. However, the method that we used to measure the anti-SARS-CoV-2 anti-spike glycoprotein-specific immunoglobulin G receptor-binding domain (s-RBD) antibodies [9] presented very good correlations with the plaque reduction neutralization test (PRNT), which is the gold standard method to assess the titer of protective antibodies in serum samples [21]. The lack of a control group of patients not responding to the third vaccination who maintained MMF therapy during the fourth dose vaccination is another limitation of the study. However, similar to what has been reported in the literature [7,8,10,12,20,22,23,24], the immunogenicity of vaccination with the fourth dose would likely have been lower than that measured in the patients enrolled in our study. The patients enrolled in our study had undergone LT for many years, and this made it possible to discontinue MMF. Conversely, discontinuation of MMF in patients undergoing a recent liver transplant may be riskier, making it difficult to replicate the results obtained in our study. In summary, suspending MMF prior to administration of the fourth dose of the anti-SARS-CoV-2 mRNA vaccine in LT recipients who failed to respond to the third dose seems feasible and safe. If confirmed in larger studies, this procedure could restore the vaccine-induced humoral and T-cell-mediated immune responses in a large portion of previously non-responder LT recipients. Although the achievement of restored immunogenicity to vaccination was not associated with a full protection from SARS-CoV-2 infection, COVID-19 occurrence in these patients was always clinically uneventful.

## Figures and Tables

**Table 1:** Main demographic and clinical characteristics of the 5 nonresponsive liver transplant recipients 6 months following the third anti-SARS-CoV-2 BTN162b2 vaccine dose. The reference intervals of biochemical parameters are reported in square brackets. Each patient is identified by a number and a corresponding color. The colors are the same as those reported in Figure 1.

	Patients				
	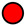 1	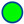 2	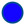 3	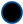 4	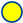 5
Sex	F	M	F	M	M
Age (years)	70	75	50	68	78
Time from LT (months)	31.8	83.9	19.0	22.5	314.4
Etiology of liver disease	PBC	HCV	AIH	AH	HBV
Immunosuppressive daily dose or drug levels					
Mycophenolate mofetil (g/day) ^§^	2	2	1	1	2
Tacrolimus (ng/mL)	4.82	4.64	6.49	4.76	3.40
Neutrophils (n ∗ 1000/µL) [2.0–7.50]	1.39	4.07	4.13	1.71	2.68
Lymphocytes (n ∗ 1000/µL) [1.0–4.0]	2.21	0.70	3.72	0.85	1.75
Platelets (n ∗ 1000/µL) [150–400]	266	204	506	114	227
AST (U/mL) [f: 4–32, m: 4–40]	20	12	23	12	16
ALT (U/mL) [f: 4–33, m: 4–41]	22	10	15	12	9
Bilirubin (mg/dl) [0.20–1.0]	0.56	0.27	1.35	0.39	0.91
Albumin (g/dl) [4.02–4.76]	4.51	4.03	3.49	4.45	4.53
eGFR (mL/min/1.73 m^2^)	46	31	62	39	33

LT: liver transplantation; PBC: primary biliary cholangitis; HCV: hepatitis C virus; AIH: autoimmune hepatitis; AH: alcoholic hepatitis; HBV: hepatitis B virus; ^§^: daily dose of mycophenolate mofetil before and after its temporary suspension; AST: aspartate aminotransferase; f: females; m: males, ALT: alanine aminotransferase; eGFR: estimated glomerular filtration rate (MDRD4 formula).

## Data Availability

The clinical and laboratory data used to support the findings of this study are included within the article.
